# Anatomical structure responsible for direction changing bilateral gaze-evoked nystagmus in patients with unilateral cerebellar infarction

**DOI:** 10.1097/MD.0000000000019866

**Published:** 2020-04-24

**Authors:** Hyung Lee, Hyun Ah Kim

**Affiliations:** a Department of Neurology; b Brain Research Institute, Keimyung University School of Medicine, Daegu, Republic of Korea.

**Keywords:** cerebellum, nystagmus, pathologic, stroke

## Abstract

The direction-changing bilateral gaze-evoked nystagmus (GEN) (BGEN) is a more specific sign for a gaze-holding deficit than unilateral GEN (UGEN) in a central lesion. We sought to clarify which cerebellar structure is responsible for the generation of BGEN compared with UGEN.

We studied 47 cases of UGEN or BGEN associated with isolated unilateral cerebellar infarction in the territories of the cerebellar arteries diagnosed by brain magnetic resonance image (MRI) from June 2007 to April 2014. To identify the structures involved in the generation of BGEN, the overlapped lesions of the BGEN group were subtracted from those of UGEN group and vice versa.

About half of the patients (25/47, 53%) showed BGEN and others showed UGEN. There was no difference in the interval from symptom onset to examination between 2 groups (1.3 days vs 2.5 days, *P* = .24). Thirty-five patients (35/47, 75%) with GEN also showed spontaneous nystagmus. Lesion subtraction analyses revealed that both of the patients with BGEN and UGEN had damage around the vermal pyramid, the uvula and the tonsil, parts of the biventer lobule, and the inferior semilunar lobule.

Midline and lower cerebellar structures are related to both BGEN and UGEN in patients with unilateral cerebellar infarction. Regardless of unilateral or bilateral, GEN may represent damage of the gaze-holding neural integrator control system in human.

## 1 Introduction

Gaze-evoked nystagmus (GEN) is occurred by the attempted maintenance of an extreme eye position probably due to a defective neural integrator.^[1,2]^ A failure of gaze-holding function by neural integrator leads to GEN with exponentially decreased slow phage velocity pattern during eccentric gaze.^[3,4]^ The direction-changing bilateral GEN (BGEN) is a more specific sign for a gaze-holding deficit than unilateral GEN (UGEN) in a central lesion and we cannot sure the origin of UGEN is neural integrator failure for some reasons. First, GEN in cerebellar lesion is from the cerebellar dysfunction as second neural integrator supplementary to physiologically leaky brainstem neural integrator. The cerebellar GEN is usually bilateral because the brainstem neural integrators have reciprocal innervation through vestibular commissure.^[5]^ Second, spontaneous nystagmus due to central vestibular disturbances usually can be seen when the eyes are close to the central position.^[4]^ However, during the recovery period after central or peripheral vestibular imbalance, nystagmus when gazing toward the healthy side can be only seen (after the disappearance of spontaneous nystagmus and nystagmus when gazing toward the lesion side, i.e., first-degree nystagmus obeying Alexander law) and this may mimic unilateral GEN. Moreover, even vestibular nystagmus has linear shaped slow phase velocity and GEN due to a defective neural integrator generally is known to show exponentially decreased slow phase velocity, it is not easy to discriminate GEN from vestibular nystagmus with slow-phase waveform because the slow-phase drift of GEN also can have a more constant-velocity (linear) waveform.^[4]^ Previous study reported that midline and lower cerebellar structures may be responsible in the generation of GEN in cerebellar lesion and these structures are part of a gaze-holding neural integrator control system, but all patients showed unilateral GEN in this study.^[6]^ Therefore, we sought to clarify which cerebellar structure is responsible for the generation of BGEN, which is a more specific sign for gaze holding failure.

## 2 Method

Between June 2007 and April 2014, we identified 47 patients with GEN from 229 patients associated with an isolated cerebellar infarction diagnosed by MRI from the acute stroke registry at the Keimyung University Dongsan Medical Center. In all patients, MRIs including diffusion-weighted images (DWI) and magnetic resonance angiography were performed shortly after the onset of symptoms (mean interval: 1.87 days, range 0–14 days). The territories of the 3 major cerebellar arteries, posterior inferior cerebellar artery (PICA), anterior inferior cerebellar artery (AICA), and superior cerebellar artery (SCA), were determined according to MR-anatomic templates, previously validated MR-anatomic templates for the diagnosis of the arterial territories.^[7]^ The vascular territory was determined by consensus between 2 neurologists (HL, HAK) who independently reviewed the MRIs. Patients with additional acute brainstem lesion (except middle cerebellar peduncle) or edema compressing brainstem were excluded.

Three-dimensional VOG (SMI, Teltow, Germany, resolution of 0.1°, a sampling rate of 60 Hz) was used to record spontaneous nystagmus (SN), GEN, smooth pursuit eye movement, and caloric responses. Asymmetry of vestibular function was calculated using the Jongkees formula, and caloric paresis was defined by a response difference of ≥25% between the ears.

**Figure 1:**
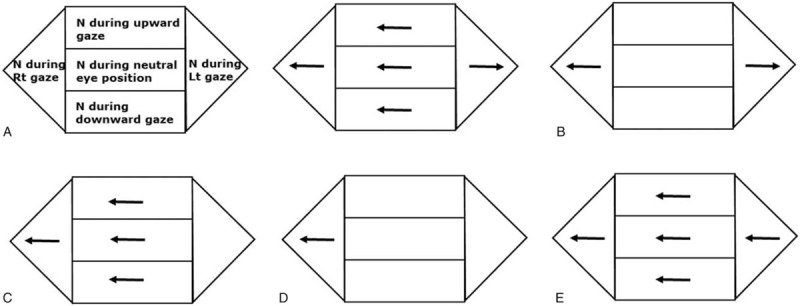
Nystagmus patterns from vestibular imbalance and/or gaze holding failure. (A) BGEN with SN, (B) BGEN without SN, (C) UGEN with SN, (D) UGEN without SN, (E) SN with direction-fixed nystagmus (vestibular nystagmus following Alexander law).BGEN = direction-changing bidirectional gaze-evoked nystagmus; N = nystagmus; SN = spontaneous nystagmus; UGEN = unidirectional gaze-evoked nystagmus. Note: The direction of arrows means the direction of fast eye movements.

All patients underwent a detailed neurologic and neuro-ophthalmologic examination performed by an experienced neuro-otologist (HL) within 3 hours after a patient's arrival at the hospital. Patients with spontaneous and direction-fixed nystagmus (i.e., the direction of nystagmus during both horizontal gaze is same with that of spontaneous nystagmus) (Fig. 1E) were excluded. BGEN defined when right-beating nystagmus initiated during gaze to the right side, and left-beating nystagmus produced during gaze to the left side with (Fig. 1A) or without (Fig. 1B) spontaneous nystagmus. UGEN defined when the only gaze to either side produced nystagmus and the other side did not initiate any nystagmus with (Fig. 1C) or without (Fig. 1D) spontaneous nystagmus.

To identify the structures involved in the generation of BGEN, the overlapped lesions of the BGEN group were subtracted from those of the comparison (i.e., UGEN) group and vice versa, revealing a percentage overlay plot. We used DWIs obtained within the first 48 hours after stroke onset and fluid-attenuated inversion recovery sequences obtained when imaging was conducted 48 hours or later for overlay technique and volume measurement. We combined the MRI images of the BGEN group and UGEN group by flipping the image of the lesions of the left brain-damaged subjects from the left to the right side. Thus all MRI images were viewed anatomically from the right side of the figure, which corresponded to the right side of the brain. Using MRIcro software (http://www.mricro.com), the lesions were mapped on slices of a T1-weighted template MRI scan from the Montreal Neurological Institute (http://www.bic.mni.mcgill.ca/cgi/icbm_view).^[8,9]^ Statistical analysis was conducted using SPSS V.21.0 (SPSS Inc, Chicago, IL) for Windows.

This study received an approval of the institutional review board of Keimyung University Dongsan Hospital.

## 3 Results

Forty-seven patients with BGEN or UGEN enrolled in the study. All were alert and oriented on admission. In most patients (38/47, 80%), acute spontaneous prolonged vertigo (>24 hours) or dizziness with nausea/vomiting was the presenting or main symptom. Mean age was 63.3 years (standard deviation [SD], 13.6). Sixty-eight percent of patients (32/47) were men. Fifty-one percent of the patients (24/47) had a lesion on the left side. Ten patients had SCA territory cerebellar infarction, 7 patients had AICA territory cerebellar infarction, and 28 patients had PICA territory cerebellar infarction. Two patients had a combined lesion in AICA and PICA territory cerebellum. The mean interval between infarction and DWI MRI examination was 1.87 days (SD, 3.27 days).

Thirty-five patients (35/47, 75%) with GEN also showed spontaneous nystagmus and they were predominantly horizontal (33/35, 94%). Horizontal spontaneous nystagmus beats away from (13/35, 37%) or toward (22/35, 63%) the side of the lesion. Eighty percent of patients (8/10) with SCA territory cerebellar infarction had spontaneous nystagmus and all but one (7/8) was ipsilesional nystagmus. All but one patients with isolated AICA territory, or AICA and PICA territories lesions (8/9, 89%) showed contralesional spontaneous nystagmus and they all had canal paresis on the side of the lesion on brain MRI. By contrast, 68% (19/28) patients with PICA cerebellar infarction had spontaneous nystagmus and 63% of them (12/19) showed ipsilesional spontaneous nystagmus. The others had downbeat (n = 2) or contralesional spontaneous nystagmus (n = 5).

**Figure 2:**
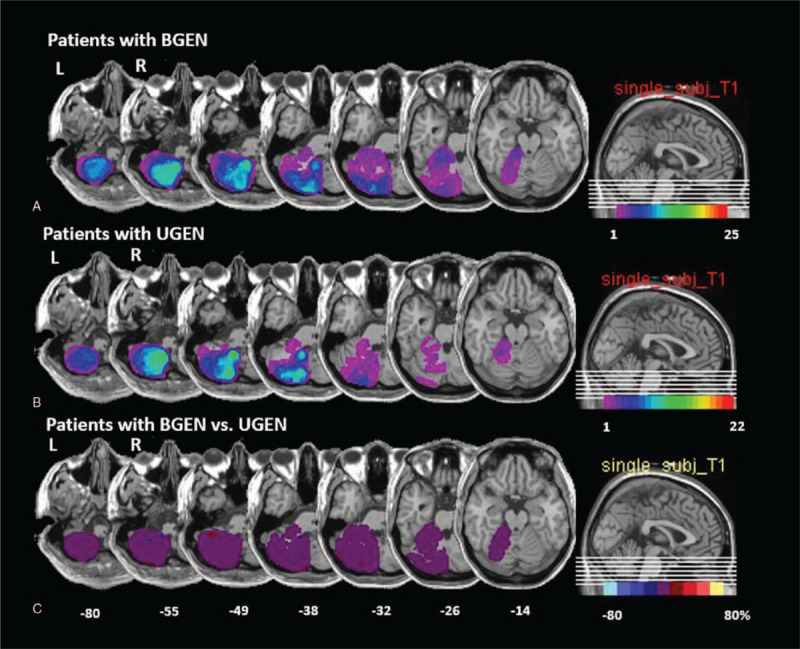
Overlapping MRI lesions in patients BGEN (A) or UGEN (B). Left-sided lesions are flipped to the right side. The number of overlapping lesions is illustrated by different colors that code for increasing frequencies, which range from violet (n = 1) to red (n = 25 in the BGEN group and n = 22 in UGEN group). Overlay plot of the subtracted superimposed lesions of patients with BGEN minus the comparison (UGEN) group. (C) The percentage of overlapping lesions of the group with BGEN after subtraction of the comparison group is illustrated by 5 different colors, where dark red represents a difference of 1% to 20% and white–yellow represents a difference of 81% to 100%. Each color represents increments of 20%. The Talairach *z*-coordinates of each transverse slice are given. This figure illustrates that there are no differences between the 2 groups. BGEN = direction-changing bidirectional gaze-evoked nystagmus; UGEN = unidirectional gaze-evoked nystagmus.

Twenty-five (25/47, 53%) patients showed BGEN and others (22/47, 47%) showed UGEN. There was no difference in the interval from symptom onset to examination between 2 groups (1.5 days vs 2.6 days, *P* = .18). Of 25 patients with BGEN, 14 patients (56%) had cerebellar infarctions in the PICA territory, 5 (20%) in SCA territory, and 4 (16%) in AICA territory. The other 2 had a combined lesion in AICA and PICA territories cerebellum. Twenty (20/47, 43%) patients had BGEN with spontaneous nystagmus and 15 (15/47, 32%) patients showed UGEN with spontaneous nystagmus. Seven (7/47, 15%) and 5 (5/47, 11%) patients showed only UGEN and BGEN without spontaneous nystagmus, respectively. All but one with UGEN showed spontaneous nystagmus beating toward the direction identical to that of UGEN. Lesion subtraction analyses of patients with unilateral cerebellar infarction revealed that there is no difference between lesions in patients with BGEN and UGEN (Fig. 2). Both of the patients with BGEN and UGEN had damage around the vermal pyramid, the uvula, and the tonsil, parts of the biventer lobule and the inferior semilunar lobule.

## 4 Discussion

Our data indicate that midline and lower cerebellar structures are related to both BGEN and UGEN in patients with unilateral cerebellar infarction. Regardless of unilateral or bilateral, GEN may represent damage of the gaze holding system in human.

Disease affecting the neural integrator causes an inadequately sustained eye position signal. This is manifest by a drift of the eyes back from an eccentric position to the central position and corrective quick phases that produce GEN. Crucial structures for horizontal gaze holding are the nucleus prepositus hypoglossi and medial vestibular nucleus; for vertical gaze holding, the interstitial nucleus of Cajal plays an important role.^[4]^ It has been suggested that a function of the cerebellum is to improve the performance of an inherently leaky neural integrator in the brainstem.^[2,10]^ Bilateral flocculus and paraflocculus were responsible for the gaze-holding deficit in the experimental studies.^[11–13]^ Unilateral flocculus lesion was more related to the vestibuloocular reflex than the gaze holding in a recent case report.^[14]^ An increment of the strong spontaneous nystagmus during gaze to the lesion side, however, suggests some involvement of the neural gaze-holding networks. Unilateral isolated tonsil lesion, which corresponds to dorsal paraflocculus in an animal, showed BGEN and this also suggests that unilateral cerebellar lesion can cause an impairment of gaze-holding system in human.^[15]^ According to lesion overlapping technique, midline cerebellar structures such as the pyramid, the uvula, tonsil, biventer lobule, and the inferior semilunar lobule contribute to the gaze-holding function in human.^[6]^ Like a previous study focused on patients with UGEN,^[6]^ our data also indicates that direction-changing BGEN, which definitely represents a deficit gaze-holding system, is associated with midline cerebellar structures.

Three-quarters of the patients with GEN combined spontaneous nystagmus in the present study. Gaze-holding mechanism itself could not be related with spontaneous nystagmus but vestibular dysfunction due to damage of adjacent vestibular structure may be responsible for spontaneous nystagmus. According to a previous study, ipsilateral spontaneous nystagmus was found more frequently with lesions in lobules of the caudal vermis, including the pyramis, uvula, and tonsil.^[16]^ It is plausible that the dysfunction of the vestibuloocular reflex caused by disruption of the cerebellovestibular connection is responsible for this ipsilesional nystagmus.^[17]^ There is a significant overlapping of responsible structures for gaze-holding and vestibuloocular reflex in midline cerebellum and this may explain frequent co-occurrence of spontaneous nystagmus and GEN in our patients.

The “leaky” neural integrator combined with vestibular damage may produce UGEN with or without spontaneous nystagmus (Fig. 1C and D),^[18]^ because centripetal ocular drift that results from the gaze-holding failure can act to oppose the vestibular tone imbalance.^[19]^ If a patient generates a slow eye movement to the left side due to vestibular imbalance, gaze to the right side results in an enhanced slow eye movement to the left side (GEN to the right side) due to centripetal drift of eye due to gaze-holding failure, while gaze to the left side results in a cancellation of the slow eye movement due to centripetal drift (no GEN to the left side).

Our study has some limitations. First, our study is a retrospective study with a small number of patients from medical records. We need to investigate a prospective study with a large number of populations. Second, because structural MRI cannot fully evaluate the functional extent of a lesion, we could not be certain that the cerebellar areas seeing intact on brain MRI were functionally intact. Third, we need a further investigation for analysis of the slow phase of the GEN using the magnetic search coil to confirm our result. Finally, because our study focused on nystagmus, further studies, including a detailed analysis of oculomotor dysfunction, are required to assess the pattern of abnormalities in saccade and smooth pursuit.

## Author contributions

Hyun Ah Kim conducted the design and conceptualization of the study, interpretation of the data, and drafting and revising the manuscript. Hyung Lee wrote the manuscript and analyzed the data.

Hyung Lee orcid: 0000-0003-0568-6104.

Hyun Ah Kim orcid: 0000-0002-2140-4763.

## References

[1] Karatas M Internuclear and supranuclear disorders of eye movements: clinical features and causes. Eur J Neurol 2009;16:1265–77. 19723293 10.1111/j.1468-1331.2009.02779.x

[2] Zee DS Leigh RJ Mathieu-Millaire F Cerebellar control of ocular gaze stability. Ann Neurol 1980;7:37–40. 6244772 10.1002/ana.410070108

[3] Leigh RJ, Devereaux MW. Advances in Understanding Mechanisms and Treatment of Infantile Forms of Nystagmus. New York: Oxford University Press; 2008.

[4] Leigh RJ, Zee DS. The Neurology of Eye Movements. Vol. 90. USA: Oxford University Press; 2015.

[5] Manto M, Manto M-U, Pandolfo M. The Cerebellum and Its Disorders. Cambridge: Cambridge University Press; 2002.

[6] Baier B Dieterich M Incidence and anatomy of gaze-evoked nystagmus in patients with cerebellar lesions. Neurology 2011;76:361–5. 21263137 10.1212/WNL.0b013e318208f4c3

[7] Tatu L Moulin T Bogousslavsky J Arterial territories of human brain: brainstem and cerebellum. Neurology 1996;47:1125–35. 8909417 10.1212/wnl.47.5.1125

[8] Rorden C Fridriksson J Karnath HO An evaluation of traditional and novel tools for lesion behavior mapping. Neuroimage 2009;44:1355–62. 18950719 10.1016/j.neuroimage.2008.09.031 PMC2667945

[9] Rorden C Karnath HO Using human brain lesions to infer function: a relic from a past era in the fMRI age? Nat Rev Neurosci 2004;5:813–9. 15378041 10.1038/nrn1521

[10] Kamath BY Keller EL A neurological integrator for the oculomotor control system. Mathematical Biosci 1976;30:341–52.

[11] Takemori S Cohen B Loss of visual suppression of vestibular nystagmus after flocculus lesions. Brain Res 1974;72:213–24. 4209081 10.1016/0006-8993(74)90860-9

[12] Waespe W Cohen B Raphan T Role of the flocculus and paraflocculus in optokinetic nystagmus and visual-vestibular interactions: effects of lesions. Exp Brain Res 1983;50:9–33. 6357831 10.1007/BF00238229

[13] Zee DS Yamazaki A Butler PH Effects of ablation of flocculus and paraflocculus of eye movements in primate. J Neurophysiol 1981;46:878–99. 7288469 10.1152/jn.1981.46.4.878

[14] Park HK Kim JS Strupp M Isolated floccular infarction: impaired vestibular responses to horizontal head impulse. J Neurol 2013;260:1576–82. 23370610 10.1007/s00415-013-6837-y

[15] Lee SH Park SH Kim JS Isolated unilateral infarction of the cerebellar tonsil: ocular motor findings. Ann Neurol 2014;75:429–34. 24812698 10.1002/ana.24094

[16] Ye BS Kim YD Nam HS Clinical manifestations of cerebellar infarction according to specific lobular involvement. Cerebellum 2010;9:571–9. 20711853 10.1007/s12311-010-0200-y

[17] Dietrichs E Clinical manifestation of focal cerebellar disease as related to the organization of neural pathways. Acta Neurol Scand Suppl 2008;188:6–11. 18439215 10.1111/j.1600-0404.2008.01025.x

[18] Jacobson GP McCaslin DL Kaylie DM Alexander's law revisited. J Am Acad Audiol 2008;19:630–8. 19323354 10.3766/jaaa.19.8.6

[19] Khojasteh E Bockisch CJ Straumann D A mechanism for eye position effects on spontaneous nystagmus. Conf Proc IEEE Eng Med Biol Soc 2012;2012:3572–5. 23366699 10.1109/EMBC.2012.6346738

